# Supplementary material for: NMR-Based Metabolomics Demonstrates a Metabolic Change during Early Developmental Stages from Healthy Infants to Young Children
Source: Metabolites. 2023 Mar 18;13(3):445. doi: 10.3390/metabo13030445 (PMC10058828; doi:10.3390/metabo13030445)
Supplement: Supplementary file 1 [file metabolites-13-00445-s001.zip › metabolites-2172425-supplementary.pdf]

Supplemental material

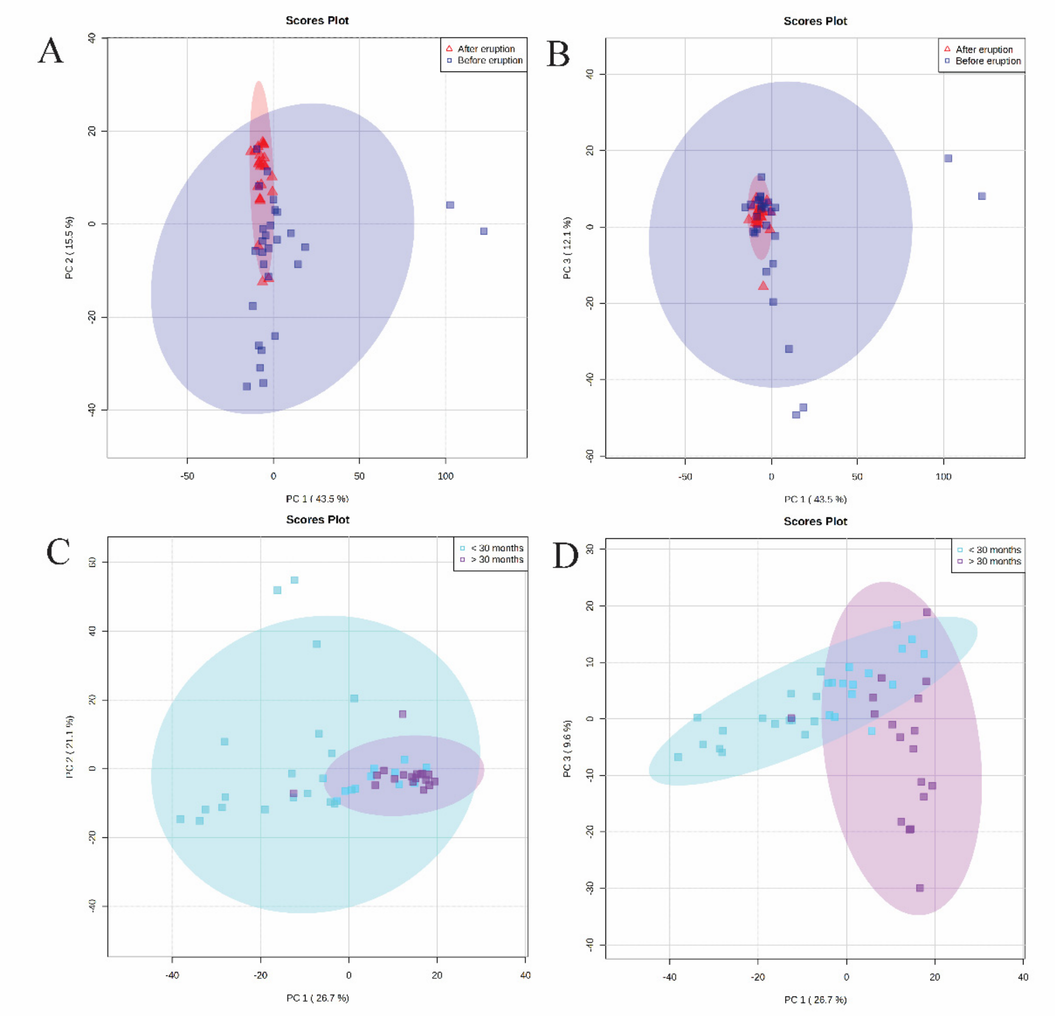

Figure S1: PCA analysis of NMR data. (A) PCA considering PC1 x PC2 and (B) PCA considering PC1 x PC3 of infants and children before and after tooth eruption; (C) PCA considering PC1 x PC2 and (D) PCA considering PC1 x PC3 of infants and children <30 and > 30 months.
